# Detailed discussion on the structure of alloy nanoparticles synthesized *via* magnetron sputter deposition onto liquid poly(ethylene glycol)

**DOI:** 10.1039/d3na00998j

**Published:** 2024-03-01

**Authors:** Mai Thanh Nguyen, Pichaya Pattanasattayavong, Tetsu Yonezawa

**Affiliations:** a Division of Materials Science and Engineering, Faculty of Engineering, Hokkaido University Kita 13 Nishi 8, Kita-ku Sapporo 060-8628 Hokkaido Japan mai_nt@eng.hokudai.ac.jp tetsu@eng.hokudai.ac.jp; b Department of Materials Science and Engineering, School of Molecular Science and Engineering, Vidyasirimedhi Institute of Science and Technology (VISTEC) Rayong 21210 Thailand

## Abstract

This paper is devoted to reviewing a decade of the development of vacuum sputter deposition onto liquid poly(ethylene glycol) (PEG) to prepare metal and alloy nanoparticles (NPs) with a controlled particle growth, size, structure, and composition. Especially, we have discussed the fine structures of alloy NPs obtained in PEG and compared them with those sputtered onto other non-volatile liquids. Finally, we have shared our prospect of applications for the resulting alloy NPs.

## Abstract 2

This paper reviews a decade of the development of vacuum sputter deposition onto liquid poly(ethylene glycol) (PEG) to prepare metal and alloy nanoparticles (NPs) with detailed discussion on the fine structure of the obtained alloy NPs.

## Graphical abstract

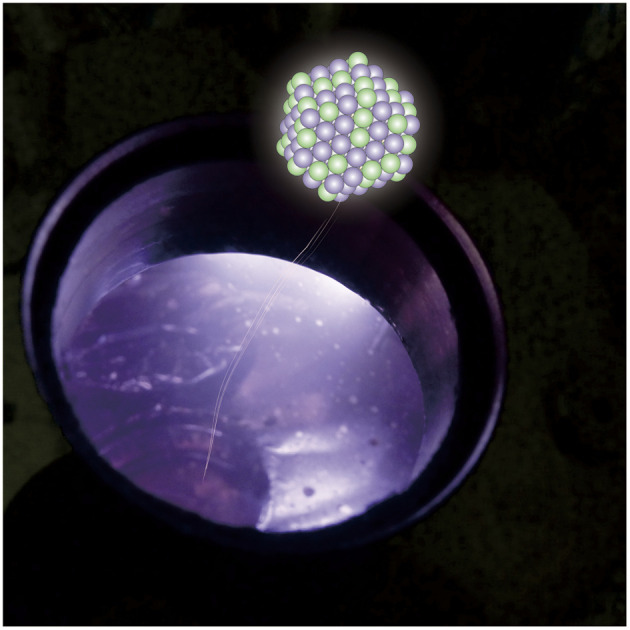

## 1. Introduction

### 1.1. Sputter deposition and alloy nanoparticles

**Fig. 1:**
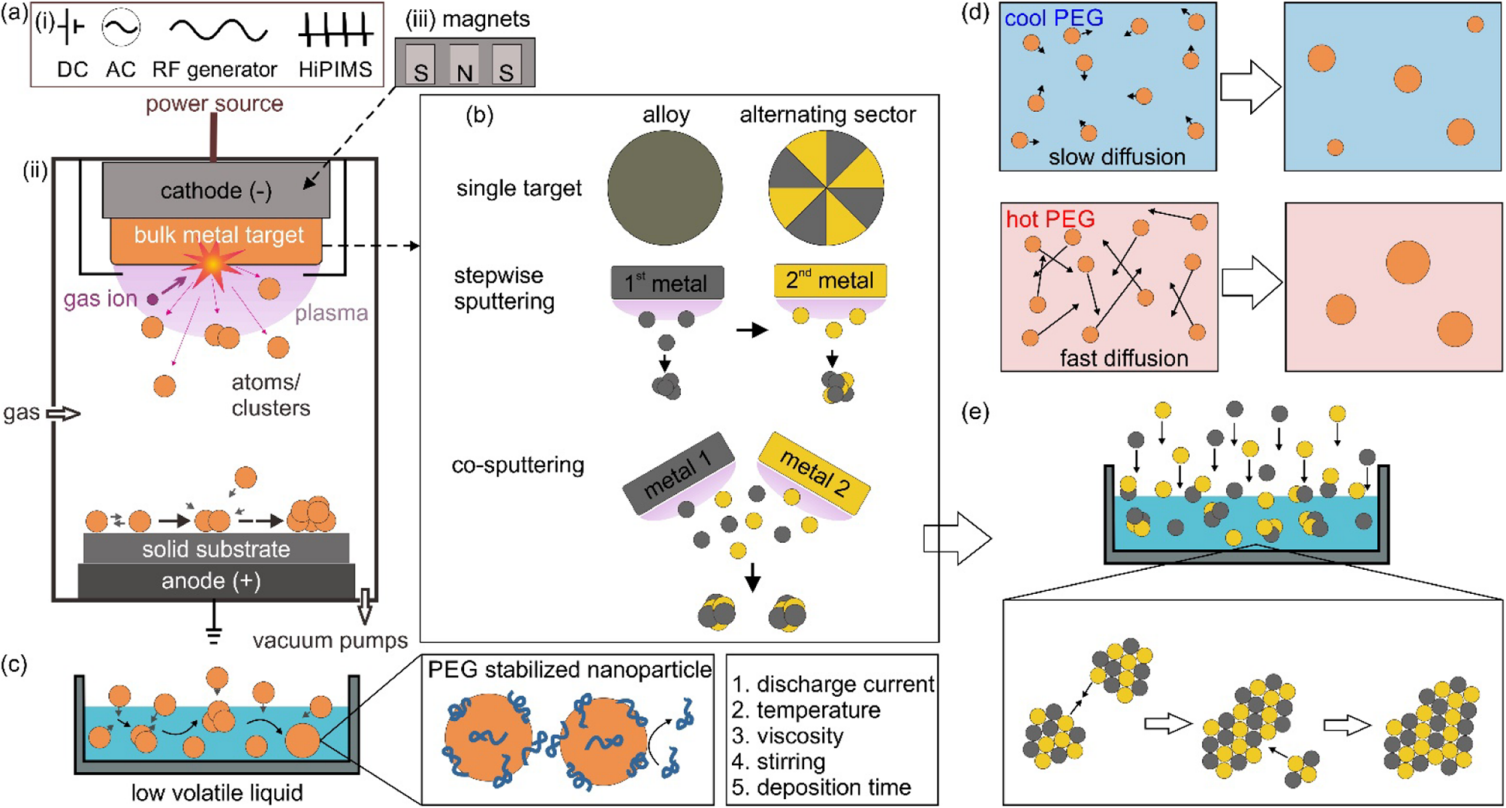
(a) General principle of sputter deposition for thin film coating on a solid surface substrate: (i) different power sources for plasma generation, such as direct current (DC), alternative current (AC), and radio frequency (RF) and high power impulse magnetron sputtering (HiPIMS). (ii) DC sputtering of a bulk metal target; (iii) in DC-magnetron sputtering, a set of magnets is placed behind the target to deflect the path of electron in plasma. (b) Different configurations of the target used in sputtering to obtain bimetallic alloy nanoparticles, including the use of single metal targets, stepwise sputtering and co-sputtering. (c) Sputtering onto liquid can be achieved by putting a low-volatile liquid, such as liquid poly(ethylene glycol) (PEG), in a container placed in the vacuum chamber. Particle growth can continue after the sputtered particles fall onto the liquid. The resulting particles can be stabilized with the adhered PEG molecules on their surface *via* steric hindrance, high viscosity of the liquid and possible static repulsive force. The main parameters involving the growth of particles in PEG include the sputter discharge current, temperature of PEG, viscosity, stirring and deposition time. (d) Particle growth in PEG under the influence of temperature. (e) Simplified illustration of co-sputtering Au and Pt onto PEG (top) with particle growth which can occur on the gas–liquid interface and inside the liquid wherein the collision and selective attachment of Au–Au sites of the sputtered clusters during growth contribute to the formation of AuPt alloy nanoparticles with Pt rich surface for stable dispersion (bottom).

Sputter deposition or sputtering is a vacuum deposition technique for making thin films and coating nanoparticles (NPs) on surfaces. In this approach, energetic gaseous ions such as Ar, Kr, and N_2_ in plasma are used to hit the surface of a metal target, resulting in the ejection of surface atoms, which then travel through vacuum and get deposited on a substrate placed in the vacuum chamber.^1,2^ Based on the power source (Fig. 1a-i) for generating and managing plasma, the sputtering method can include direct current (DC) sputtering, alternative current (AC) sputtering, radio frequency (RF) sputtering, and high power impulse magnetron sputtering (HiPIMS). Fig. 1a-ii illustrates the principle of DC sputtering. By applying a high voltage between the cathode, often located behind the target, and the anode, which is grounded, the ionization of the gas occurs and plasma is generated. Gas cations are accelerated towards the negatively charged cathode, colliding with a target and knocking off the target surface atoms. In DC magnetron sputtering (MS), magnets are arranged behind the target (Fig. 1a-iii). In the presence of a magnetic field, electrons move in a cycloid orbit instead of moving straight along the electric field. Hence, the paths of electrons are increased. Electrons collide with gas molecules more often to form denser and more stable plasma near the target surface, even at low DC voltages. This produces a higher deposition rate compared to DC sputtering at the same applied voltages and enables DC-MS to work at lower voltages. Due to a larger mass-to-charge ratio of gas ions compared with that of electrons, gas ions are less deflected by the applied magnetic field. DC sputtering is applicable for electrical conductors; however, it does not work well for dielectric materials because charge is built up at the target surface during sputtering. RF sputtering can be used for dielectric materials owing to the use of AC. In RF sputtering, plasma is produced by applying a high-frequency alternating field. However, RF sputtering has a low coating rate with possibly uneven plasma density due to the use of a large cathode. In addition, an RF generator is more expensive compared to DC power sources. HiPIMS delivers intense pulses to the cathode with a high power of kW to MW (compared with a few tens to hundred watts in DC-MS) to create extremely dense plasma, whereas a low-duty cycle is applied to prevent high heat dissipation to the cathode.^2^ In HiPMS, apart from sputtered neutral atoms, ionized sputtered atoms are also formed. Ionized atoms can increase reactivity, film density, and the coverage of high aspect ratio features. Among the discussed sputtering techniques, DC MS is the most widely used for coating, with the main advantages of stable plasma for a high sputtering rate at a relatively low vacuum and using a DC power source.

Sputter deposition can be used to synthesize NPs dispersed in a liquid by placing a non-volatile liquid in the chamber, which will be discussed in the next session. One of the immediate advantages that can be found is making bi or multimetallic alloy NPs in a straightforward approach and a reproducible manner.^3,4^ Bimetallic NPs open more room for tailoring the characteristics, properties, and functionality compared with monometallic NPs.^3–5^ In catalysts, for example, it is necessary to reduce materials used for catalysts, which are based on scarce and expensive metals such as Pt, Pd, Rh and Ru, for various practical applications.^6,7^ Making NPs of small size and high specific surface area to expose more active sites is one approach.^6^ The other is adding the second element to form bimetallic NPs to reduce the material cost on the one hand and improve the catalytic properties on the other hand.^7^ The two approaches are often combined for better performance. For example, Pt_3_Co NPs were found to outperform Pt NPs in oxygen reduction reactions, ORR, wherein Pt/C is considered a conventional catalyst.^8^ Recently, bimetallic NPs of non-precious metals could approach the performance of the conventional catalysts of precious metals.^9,10^ For example, Fe–Co–polyaniline-based catalyst exhibited high ORR activity (60 mV below Pt/C), performance stability (700 h at a fuel cell voltage of 0.4 V) and 4 electron selectivity.^9^ This can come from the novel structures that provide new properties of bimetallic NPs.^11–13^ In a high-entropy alloy, the atoms lose their elemental identity and the electronic structure of the high-entropy alloy is broadened.^14^ The idea of modifying and/or changing the electronic structure to boost the performance can be considered for bimetallic NPs; *e.g.*, pseudo-Rh in terms of electronic structure and catalytic performance in NO_*x*_ reduction or CO oxidation reactions has been reported for Pd–Ru solid solution alloy (SSA) NPs.^11–13^ However, the synthesis of bimetallic NPs can face significant challenges, which involve forming the desired structure, composition, size, and phases with high stability and homogeneity. For instance, the synthesis of binary SSA NPs in a solution can have difficulty in simultaneously reducing two metal precursors which is caused by the difference in their reduction potentials. Thus, subsequent nucleation and growth can result in a core@shell structure or segregated structure rather than a solid solution alloy. In addition, in a binary system that contains miscibility gaps, the formation of the composition in the gap is challenging. Sputter deposition of two metals does not need to deal with reduction potentials as it physically ejects atoms from the bulk metals. In addition, the alloy can be formed and trapped under non-equilibrium conditions.^15^

The metal target can be designed for making bimetallic or multimetallic alloy NPs. Fig. 1b illustrates sputtering using single targets (alloy and binary target where two metals are placed in alternating sectors), stepwise sputtering and co-sputtering of two metal targets for obtaining alloy NPs.^3,4,16^ The sputtering rate difference of two metals in an alloy target should be taken into account as the composition of the target changes over time, *i.e.*, metal with a higher sputtering rate remains more in the target.^3,4,16^ Thus, the following sputtering could not reproduce the initial sputtering experiment. On the other hand, using one target with alternative sectors of two metals would require the fabrication of different targets for varying the composition. Forming SSA NPs is generally difficult for stepwise sputtering without any further treatment (*e.g.*, thermal annealing). This is because the nucleation and growth of the first sputtered metal could readily happen before the deposition of the second metal. To a large extent, the co-sputtering is like simultaneous sputtering, whereas the composition of the sputtered NPs can be controlled by varying sputtering parameters such as applied voltage/current or location of the liquid with respect to the two (or more) targets while using two or more metal targets.^3,4,16^ This offers a quick and fine adjustment of the obtained particle composition without worrying about the different sputtering rates for different metals. The target can be sputtered to the end of its life till a hole appears. The alloying can occur in the gas, gas–liquid interface and liquid phase in sputtering onto liquid.^3,4,16^

### 1.2. Sputter deposition onto liquid poly(ethylene glycol)

**Fig. 2:**
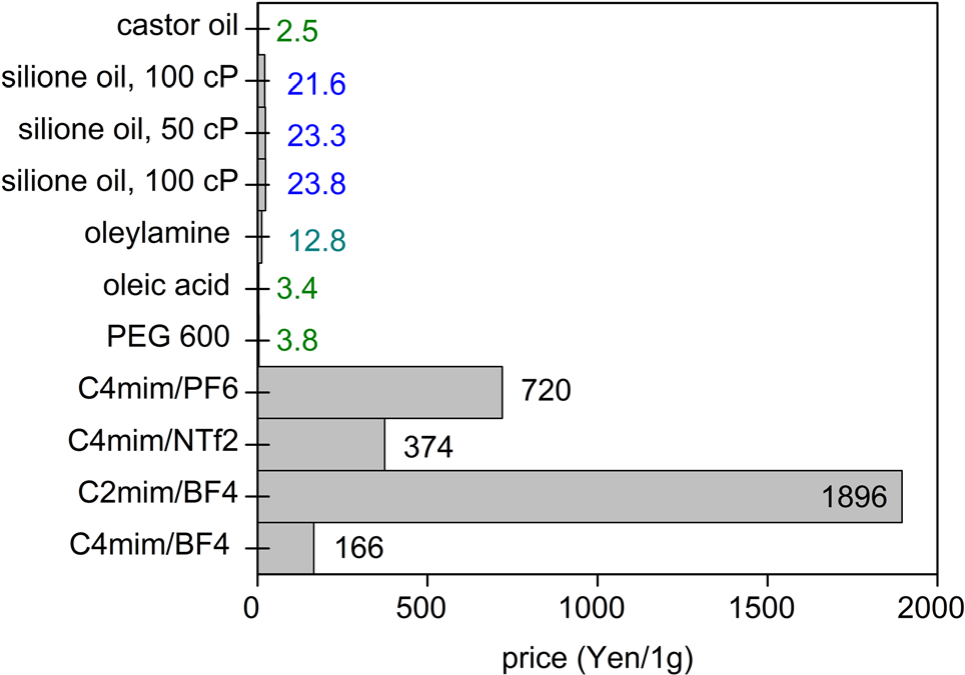
Costs of some liquids for sputtering in Japanese Yen per 1 g (as of July 2021, calculated from pricing given by Sigma-Aldrich and Wako).

When a low volatile liquid is placed in the sputtering chamber, sputtered particles can land on, penetrate, diffuse, collide and grow in the liquid (Fig. 1c). The physicochemical properties of the liquid influence the formation of particles. The method has been explored for creating NPs, especially metal NPs, well dispersed in the liquid and for fine control of the particle growth, size, shape and structures.^4,5,16^ Some pioneering works in this field include the sputtering of Ag and Fe onto silicone oil by Wagener (1999),^17^ of Au onto ionic liquids (ILs) by Torimoto (2006)^18^ for stable NP dispersion and good size control, and of Au onto molten salt for fluorescent nanoclusters by Yonezawa and Nishihara (2010).^19^ Castor oil and poly(ethylene glycol) (PEG) have been introduced respectively by Wender *et al.* (2010)^20^ and Hatakeyama *et al.* (2011)^21^ as the liquid media of cheaper cost (40–600 times), especially when compared with ILs (Fig. 2). This is critical for practical applications, such as in catalysts for energy harvesting, when a large amount of catalyst is required.^6,7^ PEG is interesting for several reasons for this application. In general, PEG is a polymer with molecular weight from 200 to tens of thousands. When the molecular weight is less than 800, PEG is in the liquid state. The increasing importance of PEG has been reviewed by Chen *et al.*^22^ They provide valuable references to emphasize that PEG is considered safe even for internal use, utilized as medical culture media, and biodegradable. PEG is widely used in drug delivery and tissue engineering. Moreover, PEG is stable to acids, bases, and chemicals such as reducing agents (*e.g.*, NaBH_4_) or oxidizing agents (*e.g.*, O_2_ and H_2_O_2_). This makes PEG a suitable medium and stabilizing agent for synthesizing NPs. In addition, owing to the benign feature of PEG, the synthesis of NPs using PEG as ligands and/or as the medium or as a part of the solvent can lead to “green synthesis”. As PEG has sufficiently low vapor pressure, it can be used as a substrate in a vacuum for the sputter deposition of metals.^23^ Besides, the high viscosity and binding function of PEG to the particle surface (with the terminal hydroxyl and abundant ether groups in the polymer chain) can effectively stabilize the sputtered species (Fig. 1c).^21,22,24^ As a polymer, PEG can have a steric hindrance effect in stabilizing NPs (Fig. 1c). The Zeta potential, *ζ*, measurement of Ag and Cu in PEG show *ζ* = −35 ± 4 mV for Ag and *ζ* = −44 ± 6 mV for Cu, suggesting Cu–PEG is more stable than Ag–PEG.^25^ Negative theta potential is supported by the calculation results that PEG attained a negative charge after interaction with the metal/oxide surface.^25^ PEG forms a low density adsorption layer on the surface of NPs with a thickness of about the characteristic size of an ideal chain, roughly 1.0 nm (PEG MW = 400 g mol^−1^), wherein the grafted PEG chain has no significant interaction. This layer contributes to stabilizing NPs in PEG (Fig. 1c). However, as the adhering layer is thin, and the fact that PEG is not a strong coordinating ligand to the metal, the thin and low density PEG layer can be insufficient to screen the van der Waals forces between nanoparticles.^25^ As a result, agglomeration has occurred.^25^

Furthermore, the use of PEG to capture the NPs allows for the subsequent dispersion of NPs in water, as liquid PEG and water are miscible in all ratios.^22,26^ This feature and the biocompatibility of PEG make PEG-coated sputtered NPs promising for bio-applications.^22,26–28^ In a study to primarily evaluate PEG grafted NPs for bio-application, water was added to the dispersion after sputtering.^29^ The NPs show good colloidal stability in water, as reported by Pišlová *et al.*, *e.g.*, *ζ*_Au–PEG–water_ ∼−66.5 mV.^29^ However, according to the authors, upon introducing some amount of salt (the exact concentration was not reported) in the culture medium, aggregation occurred, suggesting the screening effect (*ζ*_Au–PEG–water/culture-medium_ ∼ −7.3 mV). Thus, more effort should be devoted to the practical application of PEG-coated NPs in *in vivo* and *in vitro*. Some examples include further functionalizing PEG for better stability and additional surface modification (*e.g.*, addition of chitosan) of the sputtered nanoparticles.^27,30^

It is known that due to the hygroscopic property, when in air PEG adsorbs/absorbs a certain amount of water,^31^ which is often intended to be removed before sputtering by means of vacuuming. There can be some differences in properties^32^ and interaction of PEG with NPs when in a vacuum and in the air. Unfortunately, this topic has not been examined regarding the sputtered NPs. The NPs obtained in PEG are purified and analyzed after exposure to air after sputtering.

The neat liquid PEG(s) (MW = 200, 300 g mol^−1^) show Newtonian fluid behavior and their viscosities are constant with the applied share rate.^33^ However, dispersions of particles such as multiple wall carbon nanotubes (MWCNT) (even with low concentrations) and/or TiO_2_ into PEG (MW = 400 g mol^−1^) were found to show non-Newtonian pseudo-plastic behavior (less viscous when more shear is applied).^34,35^ In our sputtering onto PEG, PEG was stirred with a stirring bar placed near the liquid surface.^36^ This not only helps refresh the PEG surface but also reduces the viscosity for transporting the sputtered particle to the bulk liquid, and thus reduces the aggregation and growth of the particle caused by growth on/near the liquid surface.^36^ An average velocity of NPs in PEG under Brownian motion was calculated roughly in the order of μm min^−1^.^25^ Therefore, diffusion of NPs in PEG was considered sufficient for loading NPs to the bulk PEG during the sputtering time (in the order of some ten minutes).^25^ The viscosity of PEG decreases with increasing temperature.^21^ This can increase the diffusion and collision of particles in PEG.^21^ Therefore, the particle sizes and agglomeration of particles can depend on the temperature of PEG as the matrix. Hatakeyama *et al.* sputtered Au onto PEG (MW = 600 g mol^−1^), the temperature of which was increased from 20 to 60 °C during sputtering or from 20 to 110 °C after sputtering.^21^ They observed an increase in the size of the obtained Au NPs with increasing the temperature of PEG. In addition, the particle size is almost linearly proportional to *T*/*η* where *T* is the temperature and *η* is the viscosity of PEG during sputtering. Thus, the growth mechanism of NPs is governed by the collision frequency of the sputtered particles in PEG. However, upon comparing the *T*/*η* dependent particle size of Au obtained in PEG and in ILs and the fact that smaller NPs were found in ILs with smaller viscosity than PEG, the authors proposed that not only the collision frequency but other factors such as different stabilization mechanisms of the liquid could be taken into account. Our group found that PEG stabilizes differently to different metal elements.^16^ For example, PEG stabilizes Pt better than Au, *i.e.*, a higher adsorption energy and a shorter metal–O bonding length was found for Pt–PEG compared with Au-PEG.^37^ As a result, a smaller size and better colloidal stability of Pt and its alloy than that of Au were observed.^37^ An increase in the temperature of PEG after sputtering resulted in a slow increase in the particle size of Au for temperatures below 80 °C and fast increase in size for temperatures above 80 °C.^21^ Hatakeyama *et al.* deduced that the cohesion of small particles to form bigger particles and agglutination of large and stable NPs are accounted for the size increase below and above 80 °C, respectively.^21^ Brown *et al.* monitored the temperature of PEG (MW = 600 g mol^−1^) during 3 sputtering cycles each with 300 s.^38^ PEG could reach 150 °C after 100 s of sputtering in the first cycle and it was hotter over cycles. The highest temperature at the end of the third sputtering cycle is *ca.* 200 °C. Similar phenomena were observed, but with lower temperatures (maximum 50 °C lower), for different ILs, *i.e.*, 1-ethyl-3-methylimidazolium triflate (Emim Tf), 1-decyl-3-methylimidazolium triflate (Dmim Tf), and 1-decyl-3-methylimidazolium bis-(trifluoromethylsulfonyl)imide (Dmim Tf2N). However, the increase of particle size for Pt under the influence of temperature is not significant, *i.e.*, less than 1 nm for Pt obtained in PEG and in three ILs as compared with that observed for Au. The latter increased from 2 nm to 8 nm in PEG and from less than 1 nm to about 4 nm in IL, *i.e.*, 1 butyl 3 methylimidazolium tetrafluoroborate (BMIM BF_4_), when liquid temperature increased from 20 to 80 °C.^21^ This indicates that the degree of the impact of the temperature of the liquid on the particle size also depends on the kind of metal elements.

Recently, in an experiment with MS-driven gas condensation of Ag, Cu, and Ag–Cu onto PEG (MW = 400 g mol^−1^) combined with simulation, Biliak *et al.* reported that the kinetic energy of NPs is not enough for penetrating the PEG surface.^25,39^ Further, analysis results with X-ray photoelectron spectroscopy (XPS) indicated that the obtained Cu NPs in PEG of *ca.* 20 nm are mainly metallic with limited oxidation in comparison to more oxidation observed in a previous report.^39^ Note that in this sputtering technique, the NPs are formed and grow in the gas phase from the sputtered atoms/clusters *via* gas condensation before their soft landing on PEG. From the energy point of view, the kinetic energy upon landing on PEG of Cu NPs is 10^−2^ eV per atom, thus referred to as small for the metal atoms in NPs to stay “chemically intact” without being oxidized by the matrix.^39^ In contrast, the sputtered atoms have higher energy in the order of eV,^1,40,41^ referred to as hyperthermal particles. They could interact more with possible oxidative molecules in the liquid matrix and then become more prone to oxidation when exposed to the air.^39^

PEG was modified with plasma; *e.g.*, under Ar RF plasma treatment of the surface of PEG (MW = 400 g mol^−1^) coated stainless stain sample, PEG was proposed to undergo cross-linking.^42,43^ In this process, Ar ions (plasma) break a C–O bond and a neighbouring C–C bond. Then, the resulting dangling bonds form the propylene center for cross-linking PEG molecules. Experimentally, the C–C–C bond was detected with a high fraction in a C1s XPS spectrum of PEG (MW = 400 g mol^−1^)—stainless stain sample treated with Ar plasma, wherein it was not observed in the O_2_–Ar plasma treated one. The cross-linking of PEG was also referred to as the origin of higher water contact angle and better film stability (against dissolution of the PEG film in phosphate buffered saline) of the former sample compared with the latter one. The plasma treatment of PEG results in less hydrophilicity, which implies a polymerization of PEG under plasma.^42,43^ In DC-MS, the temperature of PEG could increase up to 200 °C.^38^ However, to the best of our knowledge, it has not been reported so far whether significant cross-liking of PEG occurs and how this could influence the binding and stabilization of PEG to the sputtered NPs.

**Fig. 3:**
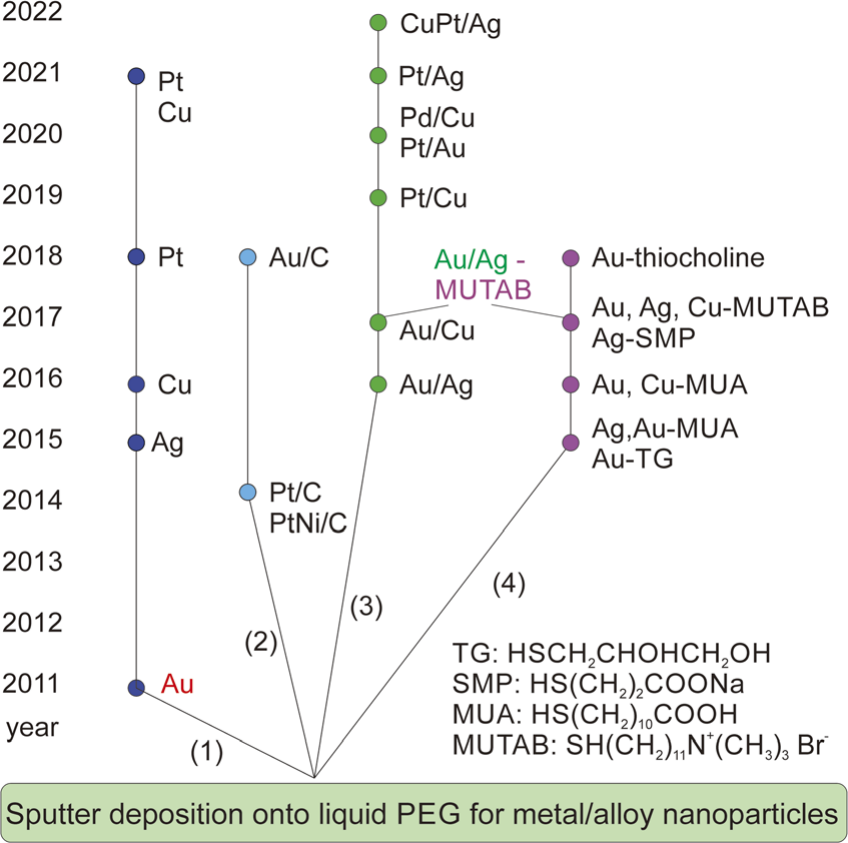
A decade of development of sputter deposition onto liquid PEG to synthesize metal and alloy NPs and nanoclusters.

The sputtering deposition onto PEG has resulted in NPs of various metals and metal alloys.^3,4,16^Fig. 3 summarizes the progress in a decade since liquid PEG was introduced into sputter deposition. The first branch is related to the sputter deposition for monometallic NPs and study the particle growth by varying the synthesis parameters such as liquid temperature (Fig. 1c), viscosity, discharge current, deposition time, storage time, and stirring.^21,27,36,44–46^ The findings show that the temperature and viscosity of PEG during and after sputtering are involved in controlling particle growth by changing the particle diffusion velocity.^21^ A higher temperature associated with a lower viscosity allows for higher diffusion velocity of particles, and hence more collision and growth of sputtered species to form bigger particles (Fig. 1d). The stirring of PEG is found to refresh the liquid surface and improve the diffusion and dispersion of the particles into liquid PEG; therefore, higher stirring speeds produce smaller and more uniform NPs.^36^ Results for Ag sputtered onto silicon oil based on *in situ* light extinction spectroscopy technique also suggests that stirring promoted the formation of smaller NPs although it did not influence the final size.^47^ By keeping the temperature of the targets and PEG unchanged, a higher discharge current resulted in bigger NPs.^45^ This can be caused by a bigger initial size of particles landing on the liquid due to more collision/growth in the gas phase when using a higher applied current. This phenomenon is not always observed when other liquid substrates such as ILs are used and/or when two or several parameters are varied over experiments or when the experiment involves bi-, tri-metal alloy NPs.^16,37,41,48^ In the latter case, the size could be associated with the composition of the co-sputtered bimetallic AuPt, *i.e.*, the increase in the applied current for both Pt and Au target to some extents results in the same particle size.^37^ This is related to a slightly Pt-rich surface formed during the course of the reaction *via* selective collision and adhesion of Au–Au sites, as illustrated in Fig. 1e.^37^ The selective attachment brings more Au to the inside of the particles and exposes more Pt to the surface.^37^

The addition of a supporter, *e.g.*, C (Fig. 3, 2nd branch)^49,50^ or various ligands (Fig. 3, 4th branch), into PEG allows for immobilization of NPs and fine control of particle size, colloidal stability, and properties, respectively.^36,44,51–56^ The former approach offers a way to separate NPs from the liquid matrix and for the NPs to be readily used as catalysts. The latter approach enables the synthesis of fluorescent-emitting metal clusters with sizes below 2 nm and their emission wavelengths depending on the metal type and the cluster size.^36,44,51–56^ The ligands could be chosen to provide either negative^36,51,52^ or positive^53–56^ charges to the resulting nanoclusters. Electron microscopy study reveals that the sputtered metal nanoclusters could be composed of smaller, agglomerated clusters from which the fluorescent emission originates.^55^ This is different from the clear crystal structure observed for chemically synthesized nanoclusters of similar sizes.^55^

So far, the above aspects have been mainly explored for monometallic NPs.^36,44,51–55^ Studies of solid solution alloys (SSAs) synthesized by vacuum sputter deposition onto PEG have been building up the 3rd branch, which is the focus of this account.^37,56–62^ In particular, we will discuss the current understanding of the fine structure of the resulting alloys in comparison to that obtained by other synthesis methods and/or by sputter deposition onto other liquids.

### 1.3 Co-sputter deposition onto PEG for the synthesis of bimetallic NPs

**Fig. 4:**
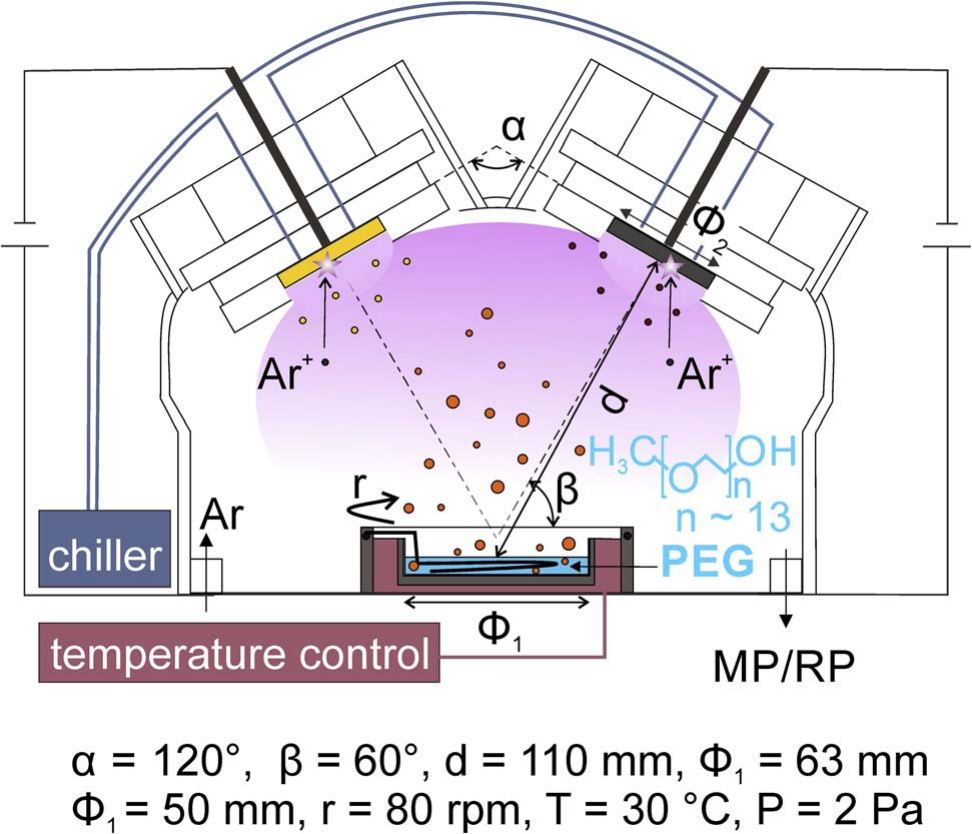
Magnetron sputter deposition onto liquid PEG for the synthesis of alloy NPs using a double-target head. Adapted with permission of ref. 58. Copyright 2018, the American Chemical Society.

Co-sputter deposition with a double-head target onto PEG has been used for synthesizing bimetallic NPs. A typical experimental set-up is illustrated in Fig. 4. Two targets are fixed to the double-target head above the liquid PEG, which is placed in a container, with the surface normal of the targets making an angle less than 90° to the horizontal plane of the liquid. The two targets are angled toward each other. The setting supports the sputtered species to collide and form an alloy. The control of the discharge currents of the two targets is independent of each other for adjusting the bimetallic composition.^37,57,58,60–62^ The cooling applied to the targets and the temperature control unit of the substrate help regulate the temperatures of the targets and liquid, respectively, which are found to have an impact on the particle size.^21,41,59^ Stirring the liquid is done with a stirrer under the liquid surface.

In co-sputtering onto PEG, the key parameters related to the particle formation are the sputtering currents applied to the targets, the temperature and viscosity of the liquid, sputtering time, stirring and the nature of the liquid. The applied currents are found to correlate linearly with the composition of the resulting alloy NPs.^37,57,58,60–62^ The temperature and viscosity of the liquid are expected to play a role in the size and agglomeration/aggregation of the NPs in liquid, for they influence the diffusion and collision frequency of particles^21,41^ in the liquid. In the synthesis NPs discussed in the next section, the temperature of the liquid was managed with the temperature control system;^37,58–62^ however, when longer sputtering was conducted, the effect of plasma heating and particle landing can make the liquid hotter or locally heated liquid surface. For monometallic sputtered NPs, it is known that stirring the liquid refreshes the liquid surface and makes particles disperse more uniformly.^36^ Such an effect is also expected in the co-sputtering. Deposition time is associated with the amount of the sputtered atoms/clusters added to the liquid for the new nucleation and continuous growth of the formed particle in the liquid.^37,59^ Thus, longer deposition time often results in a larger size and possible aggregation of NPs. In addition, it can also cause a higher temperature of the liquid medium, which in turn has an effect on particle diffusion and growth.

As for sputtering onto ILs to produce bimetallic NPs, the works by the Torimoto group are based on single targets of alternating sectors of two metals wherein their surface areas are varied to obtain different alloy compositions.^63,64^ Two or more targets for co-sputtering onto ILs have been reported by the Ludwig group.^65,66^ The formation of alloy NPs was demonstrated by both groups. The latter reported high versatility in producing various composition by co-sputtering onto ILs placed in an array of cavities in the vacuum chamber at different relative positions with respect to the two targets.^65^ The main drawback when using ILs for the synthesis of bimetallic NPs is the high cost of the liquid, which limits their practical applications requiring a large amount of NPs (Fig. 2). Furthermore, it is inferred from the Ludwig works that the purification of the sample is tough.^65^ The use of castor oil and PEG enables the sputtering with a much cheaper liquid matrix while providing “greener synthesis”, as discussed in session 1.2. Castor oil has been studied for sputtering of monometallic NPs such as Au and Ag.^20,67,68^ Using castor oil, Sergievskaya *et al.* demonstrated that the particle size increases with varying the plasma from DC-MS to HiPIMS (of higher power), whereas negligible size dependence was seen on the sputtering time and sputtering power.^67,68^ However, to the best of our knowledge, the study of alloy formed in castor oil has not been reported and hence, there is room for future researches. Using PEG as the liquid base, the alloy structure was obtained.^37,56–61^ As PEG does not tightly bind to the surface of NPs,^25^ purifying the NPs for applications, *e.g.*, catalyst, requiring bare surface can be obtained. In addition, temperature-dependent particle size is more significant for Au NPs synthesized in PEG than in ILs.^21^ This can be another factor for the size control of the alloy NPs obtained in PEG.

## 2. Crystal structures of sputtered alloy NPs

In general, binary metal systems can form SSAs with and without miscibility gaps and intermetallic compounds (hereafter, intermetallics). Phase diagrams of bimetallic systems are useful for grasping the structure in binary metal systems at a certain composition and temperature. SSAs differ from intermetallics in terms of bonding nature, structure, composition, and properties.^69,70^ Intermetallics can be considered true compounds with defined composition, structure, and properties which are often different from the metal constituents.

**Fig. 5:**
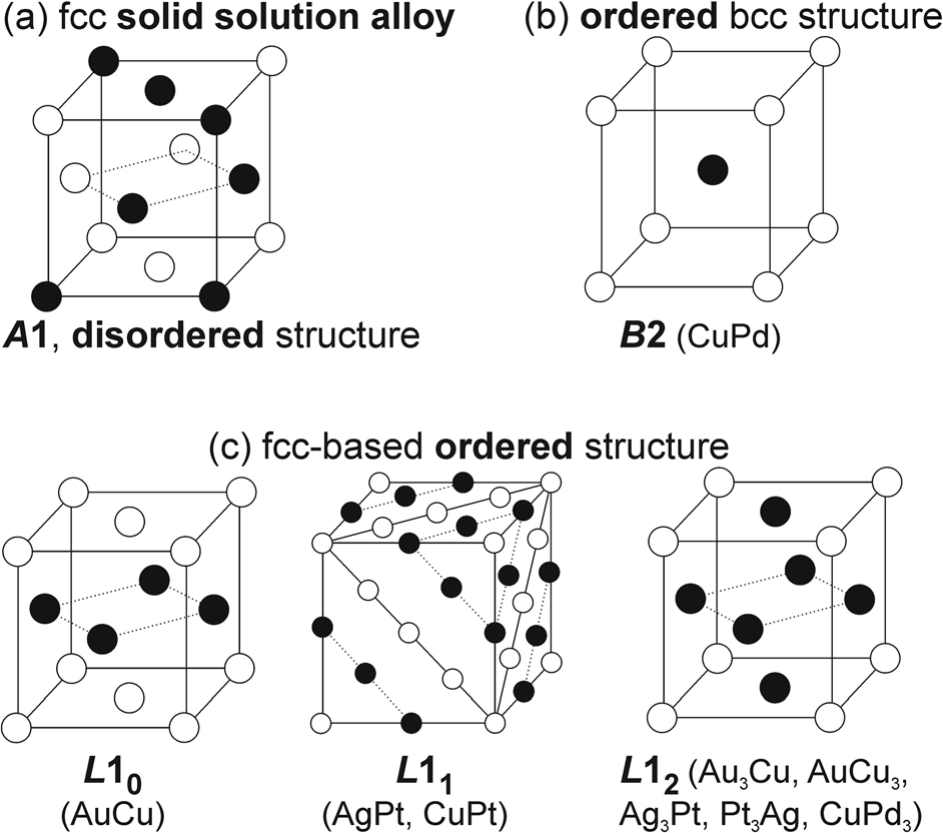
Crystal structures of (a) SSAs and some (b and c) intermetallics of *B*2, *L*1_0_, *L*1_1_, and *L*1_2_-typed ordered structures.

In the bulk phase diagram, AgAu forms SSAs in the solid state at any composition.^71^ Low-temperature phases (below 410 °C) of Au/Cu comprise some intermetallics with ordered structures (Fig. 5) such as AuCu (tetragonal structure, *L*1_0_), Au_3_Cu, and AuCu_3_ (cubic ordered structure, *L*1_2_).^72^ On the other hand, the SSAs of Au/Cu have the disordered face-centered cubic (fcc) structure (*A*1).^72^ For quenched SSAs of Au/Pt, the lattice parameter is almost linearly dependent on the composition with very slight deviation.^73^ However, the composition-dependent lattice parameters of some binary SSAs do not completely obey the straight line (Vegard's law^74^) connecting the two constituent metals.^71,72,75–78^ Negative deviations from the straight line have been reported for, *e.g.*, Ag/Au^71^ and Ag/Pt,^75,76^ whereas positive deviations have been documented for Au/Cu^72^ and Cu/Pd^77,78^ (quenched samples). These facts should be considered when discussing the structure of binary SSA NPs. This is because the small particle size affects the crystal structure, such as the shrinkage or swelling of the lattice (due to the surface stress or structural changes and/or incorporation of heteroatoms such as O and C), and the lattice parameters can depend on temperature.^79–84^ Furthermore, although low-temperature miscibility gaps exist in the bulk phase diagrams of binary systems, SSAs of NPs with compositions in the miscibility gaps or of intermetallics have been reported.^85–87^ Bimetallic NPs are produced by co-sputter deposition at room temperature under non-equilibrium conditions,^15^ hence, SSA structures and new possible structures can be expected.

Their structures will be discussed with results from the X-ray diffraction (XRD), selected area electron diffraction (SAED), spherical corrected (Cs) scanning transmission electron microscopy (STEM) for high-angle annular dark-field (HAADF) imaging, and XPS in relation to their compositions. Besides, ultraviolet-visible (UV-Vis) spectra of sputtered NPs in liquid matrix are often collected for a quick and overall assessment of the obtained bi-, tri-metallic NPs. This is especially useful and informative when one of the metal constituents has plasmonic properties in the UV-Vis range. For example, the presence of a single plasmon resonance peak between those of Au and Ag in the co-sputtered Ag/Au NPs is evidence of alloy formation rather than core/shell NPs or a physical mixture of monometallic Ag and Au NPs.^57^ The lack of the typical plasmon resonance of Au and Ag in the co-sputtered Au/Pt and Ag/Pt NPs is an indication of the formation of binary alloy NPs.^37,61^ Such UV-Vis results are also commonly observed for co-sputtered bimetallic SSA NPs obtained using other liquid matrices, *i.e.*, ILs, and for chemically synthesized SSA NPs.^63,65,88^ Because the plasmon resonance also depends on particle size, the UV-Vis results should be discussed with the information on the particle size and agglomeration state. Below, we focus on the structures of sputtered NPs from the binary metal systems that contain miscibility gaps and/or intermetallics in the bulk.

### 2.1. Bimetallic NPs with compositions in the miscibility gaps of the bulk immiscible binary system: Au/Pt

**Fig. 6:**
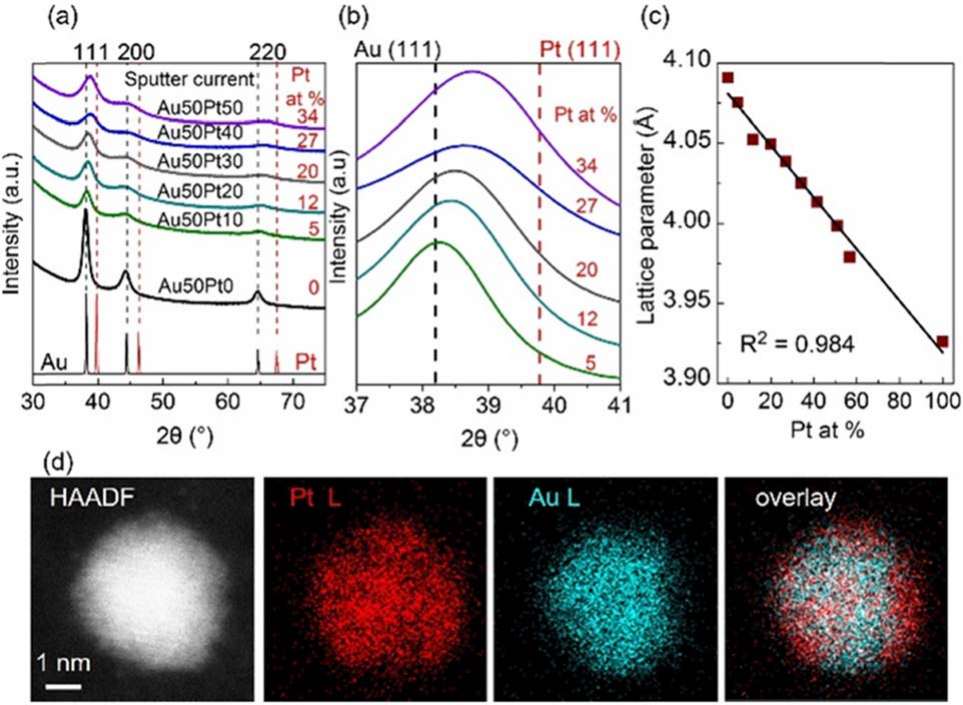
(a) XRD patterns of Au/Pt NPs using different sputter currents for Pt (0–50 mA) and 50 mA for Au target, (b) enlarged patterns of (a) for 37–41° in 2*θ*, and (c) trend of lattice parameter with composition (at.% Pt) of Au/Pt NPs. XRD reference patterns of Au and Pt are shown in black and red, respectively, at the bottom of (a) whereas dash lines in (b) mark the XRD peak positions of Au (111) and Pt (111). Compositions (Pt at. %) measured by X-ray fluorescent spectrometer are given in red in (a) and (b). (d, from left to right) HAADF image, EDS mappings for Pt L line and Au L line, and their overlay image for an Au/Pt NP synthesized by applying 50 mA to each metal target. Adapted with permission of ref. 37. Copyright 2020, the American Chemical Society.

The equilibrium phase diagram of the bulk Au/Pt contains a big miscibility gap (about 32–89 at.% Pt at 1000 °C, 15–∼100 at.% Pt at 400 °C, and 12–∼100 at.% Pt at 200 °C).^73^ The XRD patterns of the co-sputtered Au/Pt NPs at room temperature (Fig. 6a), however, reveal the fcc structure of SSAs even for compositions in the miscibility gap and no peak separation was observed (5–34 at.% Pt).^37^ The (111) peaks shifted towards Pt with an increase in at.% Pt (Fig. 6b). Meanwhile, the lattice parameter of the NPs obeys the Vegard's law (Fig. 6c).^37,73^ These results suggest that the sputtered Au/Pt NPs are SSAs. It is seen that the (111) peaks become more broadened in alloy NPs of a higher at.% Pt because a higher content of Pt correlates with a smaller particle size.^37^ This agrees with the results previously reported by the Torimoto group for AuPt SSA NPs obtained by sputter deposition of a target with an alternative arrangement of Au and Pt onto IL *N*,*N*,*N*-trimethyl-*N*-propylammonium bis(trifluormethanesulfonyl)amide (TMPA-TFSA).^64^ They show that both particle size and crystallite size decrease with a higher Pt at.%. The NPs obtained in PEG differ from those in IL, that is, (i) bigger particle sizes and (ii) the existence of the aggregation of Au-rich alloy NPs wherein the more Au, the more aggregation. Fine structures were not reported for the sputtered AuPt NPs in IL. Although similar XRD results of NPs obtained in IL and PEG were reported, and they suggest the formation of SSA,^37,64^ the fine structure analysis with HAADF imaging and elemental mapping with energy-dispersive X-ray spectroscopy (EDS) in Cs-STEM for NPs obtained in PEG shows that certain inhomogeneity in composition could present in the SSA NPs with Pt-richer on the surface than the inside (Fig. 6d).^37^ The Pt at.% is 52.47 at.% Pt at 0.5 nm from the surface and 32.63 at.% Pt at the center, whereas an average particle composition is 37.16 at.% Pt.^37^ This is to emphasize that the fine structure of the alloy NPs in the miscibility gap can be different from that is inferred from the bulk measurement, *i.e.*, XRD.

The alloy structure with a Pt-rich surface can be related to the formation mechanism of the NPs in liquid, called “selective attachment”.^37^ There is no literature for direct comparison of the diffusion within PEG of the sputtered Pt and Au. However, our observation of the sputtered dispersion over time, particle size, and simulated metal–PEG binding energy revealed that PEG binds to and stabilizes Pt better than Au. Au forms bigger NPs with aggregation in PEG, whereas Pt forms small, well-dispersed NPs. The more the Pt sputtered by increasing the sputter current applied to the Pt target while keeping the sputter current applied to Au constant, the smaller the AuPt alloy NPs and *vice versa*. This indicates that Pt content plays a part in controlling the particle size; in other words, composition is correlated with particle size. Further, the increase in electric currents for both Pt and Au targets (10–50 mA (ref. 37)) did not likely alter the particle size, suggesting that with the same composition, to a certain size, particles stop growing and are stable (under the time frame of the experiment and analysis). Based on these results, we suggest that particles in PEG can grow *via* collision and adhesion of Au–Au sites from different AuPt clusters. This brings more Au to the inner part and takes more Pt to the surface (Fig. 1e). Note that PEG binds to Pt more strongly than to Au, and less effective collision (collision results in adhesion) can occur between particles when the surface is Pt-rich. Thus, the particles can grow to a certain size and surface composition, making them stable in PEG.^37^ Note that using similar experimental conditions, the particle size of single metal NPs increases when the currents increase in the range of 5–50 mA.^37,45,58^ Thus, alloying of two metals has an impact on the particle size, and such an impact is an outcome of their formed structure.

### 2.2. Bimetallic NPs with compositions of the intermetallics in bulk binary systems: Ag/Pt, Au/Cu, Cu/Pd, and Cu/Pt

**Fig. 7:**
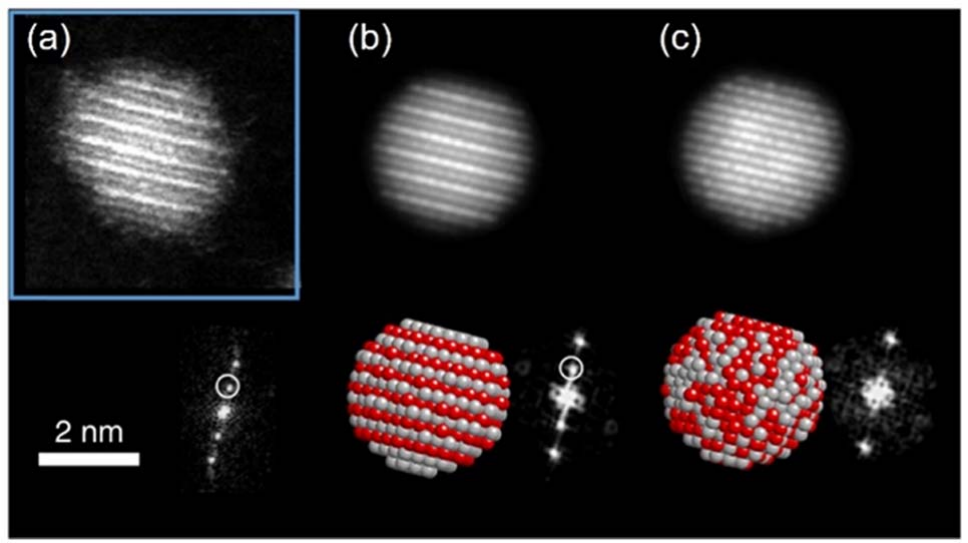
(a) STEM-HAADF image of one Ag/Pt NP oriented along the [011̄] zone axis showing *L*1_1_ phase with alternating planes of Ag and Pt and its corresponding FFT showing the superstructure reflections (marked with white circle). In the HAADF image, Pt atom columns are brighter than Ag atom columns, for Pt has a higher atomic number than Ag. (b and c) Simulated STEM-HAADF images along [011̄] zone axis for an *L*1_1_ structure and a random solid solution, respectively. Adapted from ref. 97 under CC BY license @ 2019, the Author(s). Copyright 2019, Springer Nature.

In low-temperature ranges of the bulk solid-state binary phase diagram of Ag/Pt,^89–91^ Au/Cu,^72^ Cu/Pd,^77,78^ and Cu/Pt,^92^ aside from miscibility gaps, some intermetallics have been reported. The intermetallics in Table 1 mainly have some ordered structures, that is, *B*2, *L*1_1_, *L*1_2_, and *L*1_0_ (Fig. 5). The ordered structures can be inferred from the physical property measurements (thermal/electrical conductivity, magnetic anisotropy, *etc.*),^93–96^ presence of superlattice peaks (diffraction) in XRD pattern (and/or electron diffraction, ED)^93–95^ or they can be observed directly using STEM-HAADF imaging with the *Z*-contrast of metal constituents along a certain suitable crystal direction (Fig. 7).^97,98^

**Table 1:** Phase and composition of some binary alloys

Bulk				Sputtered NPs in PEG
Phase^a^	Composition^b^ (at.%)	Structure	*T* (°C)	Composition^b^ (at.%)
Ag/Au^71^ (Ag/Au)	0–100	*A*1		0–100 (ref. 57)
Au/Cu^72^				0, 25, 35,40, 45, 55, 65, 70, 100 (ref. 58)
(Au,Cu)	0–100	*A*1	910	
Au_3_Cu	10–38.5	*L*1_2_	240	
AuCu	42–57	*L*1_0_	385	
AuCu_3_ (I)	67–81	*L*1_2_	390	
Au/Pt^73^				5, 8, 9, 12, 16, 20, 22, 26, 32–35, 41–51, 57, 63, 71, 79, 82 (ref. 37)
(Au)	0–∼12	*A*1	400	
(Pt)	∼100	*A*1		
*Au*_*3*_ *Pt*	5–40	*L*1_2_		
*AuPt*	50	*L*1_0_		
*AuPt*_*3*_	75	—		
Ag/Pt^91^				(0–100) 19.8 ± 12.2; 60.2 ± 16.2; 85.1 ± 14.0 (ref. 61)
(Ag)	0–12	*A*1	400	
(Pt)	98–100	*A*1	400	
AgPt	∼50	*L*1_1_	400	
Cu/Pd^77^				
(Cu,Pd)	0–100	*A*1	>600^c^	(0–100) 66 ± 3; 74 ± 2; 77 ± 3; 84 ± 5; 89 ± 2 (ref. 60)
Cu_3_Pd (α′)	∼7.6–22	*L*1_2_		
CuPd (β)	∼36–47	*B*2		

a *Metastable*; (solid solution alloy); M_*x*_M′_*y*_: bimetallic.

b Counted for the second metal; temperature of liquid matrix of 30 °C; where it is available, compositions measured by STEM/TEM-EDS were listed for the sputter NPs.

c Below melting temperature.

Crystal structure assessment based on the bulk physical property measurement and powder XRD analysis of the sputtered NPs is not always possible due to insufficient amount of materials. This is caused by small particle sizes and difficulties in separation of a small amount of sputtered NPs from the liquid matrices.^61^ Thus, the discussion of ordered structures based on the physical properties of NPs is not available as it was reported for thin films or bulk materials. Consequently, judging the crystal structure (SSA, intermetallic, segregated structures) of the sputtered NPs in liquid has been mainly based on the ED and TEM data and some time from XRD analysis.

**Fig. 8:**
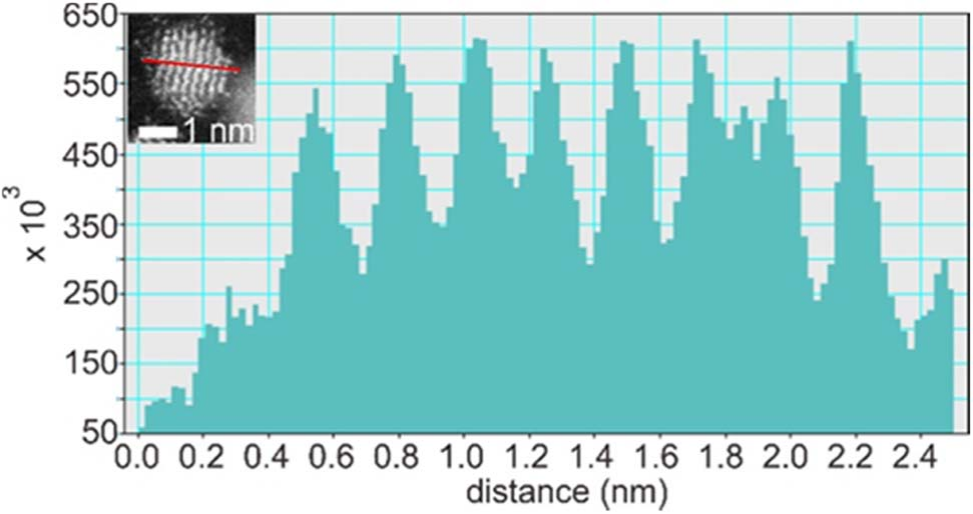
Line profile through (111) planes (marked by a red line in the inset) of an Ag/Pt NP shown in the inset of the figure. Adapted with permission of ref. 61. Copyright 2021, the American Chemical Society.

Suzuki *et al.* could separate the sputtered Cu/Au NPs from IL and collect the XRD patterns for NPs before and after increasing the thermal treatment of IL-containing NPs.^99^ Their results revealed the formation of SSA at room temperature and intermetallic Cu/Au NPs at elevated temperatures, wherein a superlattice peak identical to the *L*1_0_ structure was visible.^99^ We could collect XRD patterns of the sputtered Ag/Pt, Cu/Pt NPs in liquid PEG (30 min deposition) and compare them with that of NPs sputtered on solid substrate (glass) for 2–5 min.^59,61^ No thermal treatment was performed on the samples during and after sputtering. More peak broadening was observed for the sample sputtered onto PEG due to the smaller size of the NPs obtained in liquid substrate compared with the thin metal film on the glass.^61^ The XRD patterns of the sputtered Ag/Pt NPs and solid film on glass show fcc structure for binary compositions in the miscibility gaps and at the compositions of the intermetallics.^61^ Linear trend in composition-dependent lattice-parameter calculated from the XRD peak position was likely observed for Ag/Pt sputtered on a solid substrate.^61^ This differs from a negative deviation from the straight line observed in the bulk SSAs (quenched samples) of Ag/Pt.^75,76^ However, it is noted that for each experimental data point, a relatively large deviation (to either the negative or the positive side) from the linear line was observed for samples of Ag/Pt NPs sputtered onto liquid PEG.^61^ Thus, HAADF analysis was conducted to verify whether the structures of NPs are SSAs or segregated or ordered structures. The possible formation of an ordered structure was considered because of the following reasons. During sputtering, PEG was placed in a Petri dish, which is in contact with a metal holder and temperature control system (for cooling down and heating up). However, the surface of PEG in contact with plasma can be temporarily heated over the setting temperature.^38^ As shown by Brown *et al*. for standstill PEG, the temperature can reach 200 °C after 300 s in the third cycle of sputtering.^38^ The high temperature can induce a structure transition in Ag/Pt nanoclusters/nanoparticles and possibly form an ordered structure. This transition was observed by Pirat *et al.* when heating disordered Ag/Pt at 400 °C.^97^ The HAADF-STEM analysis with an example of an intensity line profile obtained from a HAADF image is shown in Fig. 8 for an Ag/Pt NP. The intensity ratio was calculated. If *L*1_1_ structure of AgPt exists in the NP, it can be observed with alternative layers of Ag and Pt for (111) planes (Fig. 5c), which then translates, respectively, into darker and brighter lines of atoms in the HAADF image (Fig. 7b). However, across (111) planes, no clear alternative arrangement of atom columns of Pt (high intensity, look brighter) and Ag (lower intensity, look darker) was observed for Ag/Pt NP.^61^ The result reveals that atoms of both constituent metal elements are mixed in each atom column of the NP. This was also observed in other NPs, which aligned with the zone axis parallel to (111) planes, suggesting the formation of SSAs in sputtered bimetallic NPs.^61^ Similarly, the HAADF images of the sputtered Au/Cu and Cu/Pd, Cu/Pt NPs were also observed with relatively uniform intensity for atom columns across the NPs, suggesting random distribution of both metal atom constituents in the atom columns.^58–60^ The SSA structure was furthermore confirmed with the elemental mapping and/or area analysis of the NPs wherein a relatively uniform distribution of both metal elements was observed in the NPs. The existence of miscibility gaps for the bulk AgPt and other binary systems suggests that thermodynamically, the SSA or intermetallics with composition in the gaps are unstable under equilibrium conditions. The obtained SSA NPs can be viewed as a result of a kinetic trap under non-equilibrium conditions of sputter deposition.^15^

**Fig. 9:**
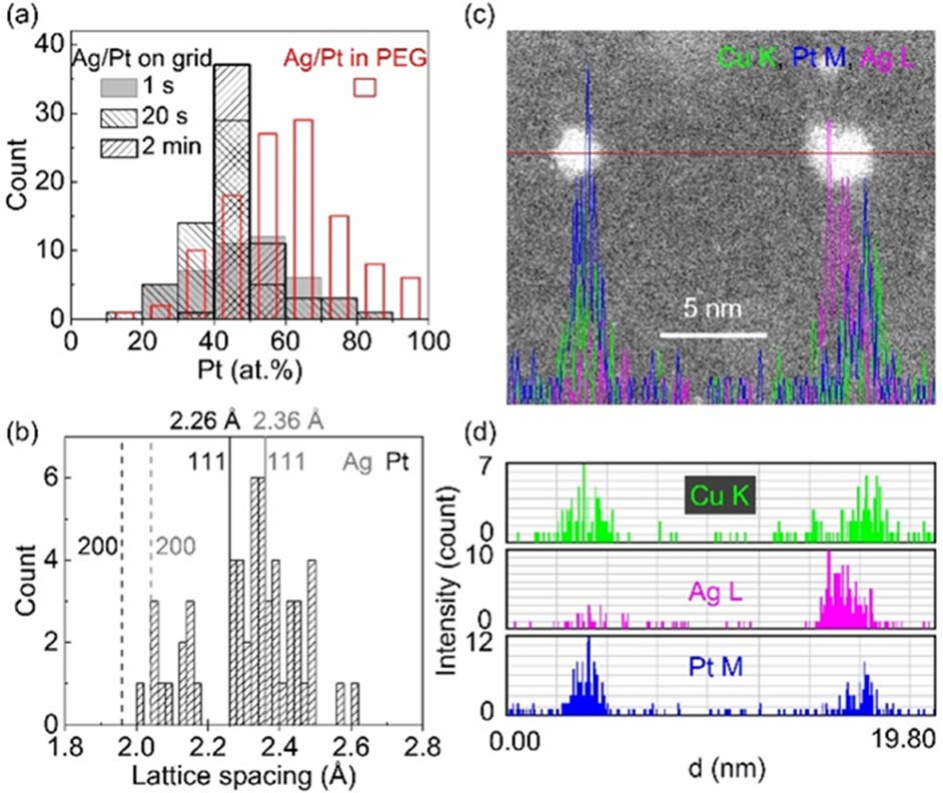
(a) Composition distribution of Ag/Pt NPs sputtered using 50 mA for both Ag and Pt targets onto (blank, red border) PEG for 30 min and Ag/Pt sputtered onto TEM grid for different times ((filled, grey, borderless) 1 s, (sparse, grey border) 20 s, and (sparse, black border) 2 min). (b) Lattice spacing distribution in SSA Ag/Pt NPs sputtered onto PEG shown in (a). Adapted with permission of ref. 61. Copyright 2021, the American Chemical Society. (c and d) EDS elemental line profiles of co-sputtered AgCuPt NPs in PEG at the position marked by red line crossing NPs in the HAADF image shown in (c). Color codes: green, purple, and blue are for Cu K, Ag L, and Pt M lines, respectively. Adapted with permission of ref. 62. Copyright 2022, the Royal Society of Chemistry.

In our study, we observed a Gaussian-like distribution of composition (Fig. 9a) among sputtered alloy NPs in PEG with the same nominal composition rather than a single composition.^61^ This indicates that the alloying occurs relatively random *via* collision and combination of Ag and Pt atoms/clusters, which is correlated with a distribution of lattice spacings of NPs (Fig. 9b). Moreover, the composition distribution of NPs in PEG (30 min sputter) is broader than that on a grid, which becomes narrower over short times of deposition (1 s to 2 min). Thus, PEG hinders both particle growth and uniformly alloying for achieving a single (uniform) composition and crystal structure. This is to say that in addition to the overall structure of the whole samples of NPs inferred from XRD data, fine structure analysis is crucial for discussing the structure of the co-sputtered NPs onto PEG. The composition distribution of NPs in a wide range for a nominal composition was also observed in co-sputtered trimetallic NPs using an Ag target and a CuPt alloy target.^62^ Although the XRD data suggests an SSA structure, the EDS line profile and HAADF analysis reveal that certain segregation/heterogeneous structures could occur in the sputtered NPs.^62^ As can be seen in the EDS line profile (Fig. 9c and d), Cu, Ag, and Pt distribute relatively uniform in the NP on the left but become segregated in the NP on the right of Fig. 9c, forming Ag and CuPt rich regions in the NP.^62^ Again, this suggests the random collision and alloying of NPs in PEG, which could contribute to the observed inhomogeneity in the composition of the trimetallic NPs. Cai *et al.* claimed a uniform distribution of Au, Pd, and Pt trimetallic NPs with STEM-EDS mapping for sequential sputter deposition of PdAu and Pt onto IL-containing carbon nanotubes.^100^ However, high-resolution EDS mappings for single AuPdPt NPs were not reported to judge the conclusion.^101^

### 2.3. Electronic structure of sputtered alloy NPs

**Fig. 10:**
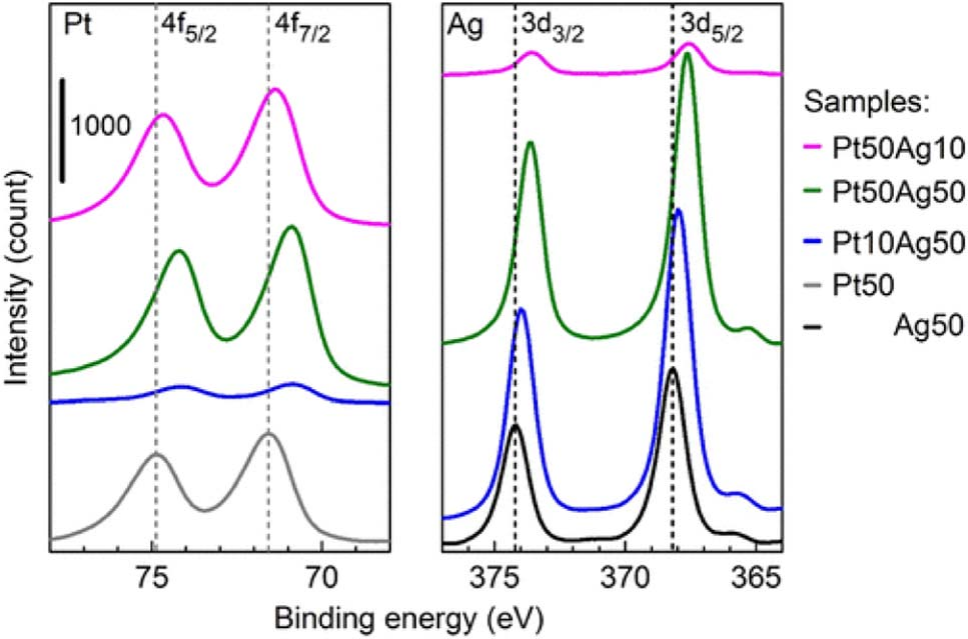
XPS spectra of Ag/Pt NPs sputtered on Si wafer for 10 s. Dashed lines refer to the reference peak position. Reproduced with permission of ref. 61. Copyright 2021, the American Chemical Society.

SSAs are known to have controllable electronic structures by varying the composition of the constituents. XPS allows studying the overall electronic structure of NPs and helps infer whether the resulting bi-/tri-metallic NPs are alloys. Because XPS is surface sensitive and co-sputtered NPs are of few nanometers, the XPS spectra can be used to study the electronic structure of the sputter NPs (not limited to the surface state). The shifts of the XPS peak were observed in co-sputtered bimetallic NPs compared with that of monometallic ones.^60,61,101^ Furthermore, the shifts vary with the composition. This is in line with the phenomenon observed for the diluted bulk alloy. An example is shown in Fig. 10 for sputtered Ag/Pt. We found that XPS spectra of Ag/Pt have Ag and Pt shifted more negatively than pure Ag and Pt, suggesting Ag becomes more positively charged and Pt becomes more negatively charged. This can be related to the electron transfer from Ag to Pt, indicating the formation of Ag/Pt alloy. The shift for Ag (Pt) becomes less when the Ag (Pt) content increases. This is reasonable because there is less dilution of Ag (Pt) by Pt (Ag) in the SSA lattice. The positive shift for Pt and negative shift for Au with the values increasing for higher Pt content were observed by Torimoto, suggesting the electron transfer from Pt to Au.^101^ XPS results are consistent with the increase in electronegativity in the order of Ag, Pt, and Au.

## 4. Conclusion and perspective

PEG shows its capability of capturing and hindering the growth of the sputtered particles to certain extents. PEG stabilizes NPs *via* binding their functional group to the particle surface, steric hindrance, and possibly certain electrorepulsive forces. The stabilizing effect of PEG is different for different kinds of metal elements. This can be understood by the difference in size and colloidal stability of different metal NPs obtained in PEG. This becomes important when discussing the formation of alloy NPs. In addition, the different stabilizing power of PEG to different kinds of metal elements and high viscosity, to a certain extent, result in the composition inhomogeneity among NPs and in single NPs. The bimetallic alloy formation in PEG is achieved for various binary systems. The formation of SSA NPs over segregated structures, intermetallics, or physical mixtures of monometallic is suggested by the overall analysis of the sputtered bi-/ternary metal alloy NPs with techniques such as UV-Vis, XRD, and XPS results. The fine structure assessment with a Cs-STEM reveals a certain inhomogeneity in the composition and structure within single NPs and among NPs. The STEM-EDS results also indicate a range of compositions for the alloy NPs obtained by sputter deposition. These observations are important as they are related to the alloy formation in PEG and should not be neglected when considering the structure of co-sputtered NPs of binary systems with miscibility gaps and/or intermetallics. The fine structure can also be crucial for understanding the performance of NPs in certain applications. Our primary results indicate the sputtered AgPt alloy NPs outperformed the Pt ones. However, mechanism and structural changes can be complicated, and further study is necessary to shed light on this matter.

The downsides of sputter deposition onto PEG include:

(i) The uniformity of particle size and colloidal stability of NPs obtained in PEG is generally less than that in ILs (exceptions can be found for some cases, *e.g.*, Pt). This can arise from the weaker stabilizing capability of PEG to NPs than that of ILs to NPs.

(ii) A relatively large composition distribution for bimetallic nanoparticles in the same bath was observed for those sputtered onto PEG, as it is also observed in some reports when ILs were used. Narrowing such distribution can be beneficial for studying such as correlation between size/composition and properties. We want to comment on this point that perhaps such an analysis was not conducted in some reports for sputered NPs obtained with other liquids. The growth of nanoparticles in PEG, which is influenced by its high viscosity and different binding with different metal atoms, can account for the composition distribution. They prevent the atoms/clusters from different elements in PEG immediately and homogeneously collide and grow.

Future directions can include the formation mechanism of SSA NPs with key questions regarding the extent to which the alloying takes place in the gas phase, gas–liquid interface and/or in the bulk liquid, how to narrow the composition of the obtained alloy NPs to a single composition for a set of experimental conditions and how to control the size separately from the particle composition. In addition, the interaction and binding of PEG to NPs of mono-, bi- and multi-metallic SSA NPs is of interest, especially in a quantitative sense. Evaluating the performance and structure stability of alloy NPs obtained in PEG is needed. The optimal amounts and maximum concentration for efficient performance can be addressed to understand the capability of the method for application purposes. The other aspect is the stability of the SSA NPs as they are formed in a non-equilibrium process near room temperature and are kinetically trapped in the SSA structure. How this SSA structure changes under applications, such as for catalysts, is of great fundamental interest. Finally, addressing the fine structure aspect and how to achieve high uniformity in size and composition can be challenging and interesting when it comes to the sputtered alloy nanoclusters or when the 3rd, 4th, and more metal elements are added to the NPs. These NPs are promising for advanced applications in optics and catalysis.

## Conflicts of interest

There are no conflicts to declare.

## Supplementary Material
